# Effects of Lithium Treatment in Pediatric Patients with Conduct Disorder: A Systematic Review and a Single-Arm Meta-Analysis

**DOI:** 10.1192/j.eurpsy.2025.437

**Published:** 2025-08-26

**Authors:** D. S. Lima, L. R. Campos, G. D. L. Dantas Henrique, A. V. de Vasconcelos, L. M. Barbosa, B. G. Fragoso Dantas, A. B. Cavalcanti Petrucci, A. L. Lima Larcipretti, G. C. Carpi

**Affiliations:** 1 Health Sciences, University Franciscana, Santa Maria-RS; 2 Health Sciences, University of Ribeirão Preto, Ribeirão Preto-SP; 3 Health Sciences, Federal University of Paraíba, João Pessoa-PB; 4 Health Sciences, Afya College of Medical Sciences of Santa Inês, Santa Inês - MA; 5 Health Sciences, Federal University of Minas Gerais, Belo Horizonte-MG; 6 Health Sciences, University Center of João Pessoa, João Pessoa-PB; 7 Health Sciences, Federal University of Ouro Preto, Ouro Preto-MG; 8 Health Sciences, Porto Alegre Clinics Hospital, Porto Alegre- RS, Brazil

## Abstract

**Introduction:**

Conduct disorder (CD) is a behavior disorder mostly presented during childhood and adolescence, with a lifetime prevalence between 2 and 10%. Atypical antipsychotics are considered the most effective pharmacotherapy for CD, nonetheless, they are associated with sedation and extrapyramidal side effects. The pediatric use of lithium is well-documented for managing bipolar disorder; however, its efficacy in treating conduct disorders in children remains uncertain.

**Objectives:**

In this study, the authors evaluate the adverse effects of lithium in pediatric patients with conduct disorder.

**Methods:**

We systematically searched PubMed, Embase, and the Cochrane Library for randomized controlled trials and controlled observational studies in pediatric patients with conduct disorder, following the PRISMA protocol. The search strategy included the following keywords: “Pediatric Population”, “Conduct disorder”, and “Lithium”. Review Manager 5.4 and Inverse Variance Random Effects were used for statistical analysis and heterogeneity was examined with the Cochran Q test and I² statistics.

**Results:**

From the search of the databases, 1.258 articles were found. After removing duplicates and ineligible studies, 3 articles were included in this study according to the inclusion criteria. We included 161 patients from 3 non-randomized trials. (Figure 1) A total of 136 (84.5%) patients were male. The mean ages between studies ranged from 5.2 to 14.2 years. Only the outcome of adverse effects related to the study population could be found in the 3 studies, and this was used to carry out the single-arm meta-analysis. The pooled proportion of adverse events was 68% (95% CI; 0.30-1.00; I² = 98%; p < 0.01). (Figure 2) Adverse events included: decreased appetite, stomachache, nausea, vomiting, headache, dizziness, pallor, and fatigue, among others.

**Image 1:**

**Figure:**
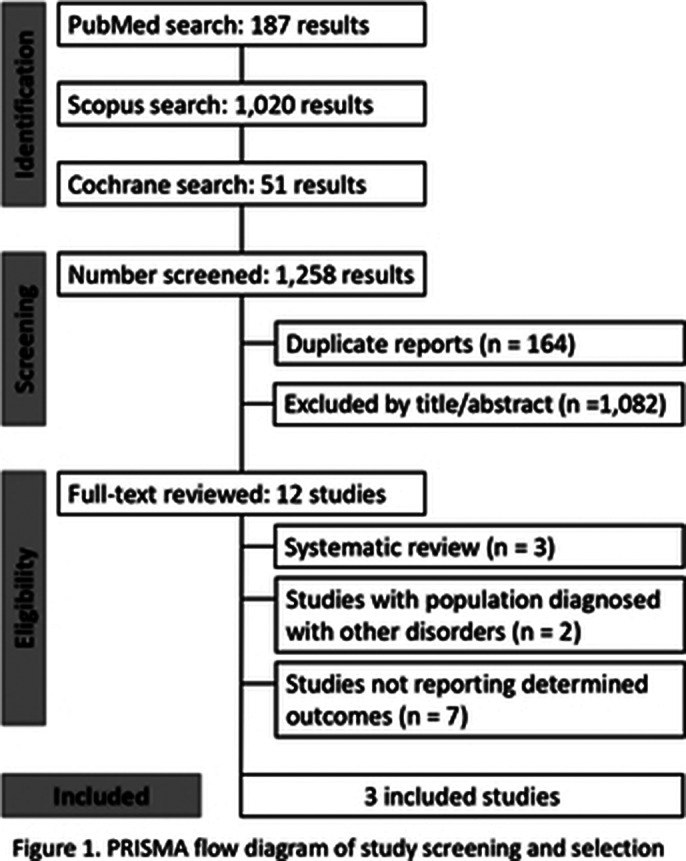

**Image 2:**

**Figure:**
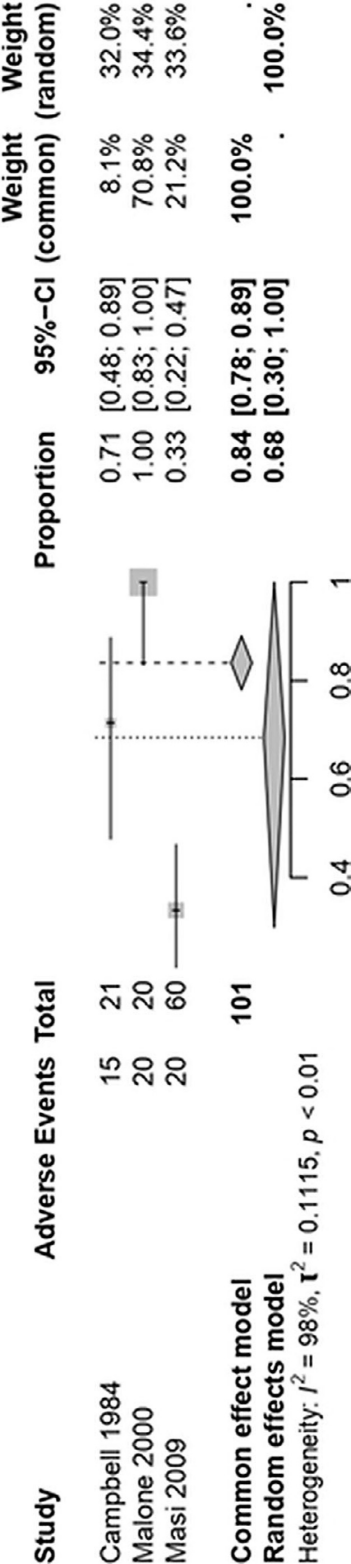

**Conclusions:**

In pediatric patients, lithium appears to be a viable treatment option for conduct disorder. However, some patients may experience adverse events such as decreased appetite, stomach pain, nausea, vomiting, headache, among others. Therefore, more comparative studies are needed to validate the safety and efficacy profile of lithium in this population.

**Disclosure of Interest:**

None Declared

